# Dreadful infectious disease outbreaks threaten flood-ravaged pakistan: short communication

**DOI:** 10.1097/MS9.0000000000000754

**Published:** 2023-05-03

**Authors:** Omer Ahmed Shaikh, Maliha Rahim, Manisha Essarani, Soeba Nadeem, Sidhant Ochani, Md. Al Hasibuzzaman, Kaleem Ullah

**Affiliations:** a Department of Medicine, Ziauddin University; b Department of Medicine, Jinnah Sindh Medical University, Karachi; c Department of Medicine, Khairpur Medical College, Khairpur Mir’s; d Department of Liver Transplantation, Pir Abdul Qadir Shah Jeelani Institute of Medical Sciences, Gambat, Pakistan; e Institute of Nutrition and Food Sciences, University of Dhaka, Dhaka, Bangladesh

**Keywords:** Child health, community health, flood, infectious diseases, public health

## Abstract

A rise in the incidence of water-borne, communicable illnesses, and viral outbreaks in Pakistan follows periods of heavy rainfall. Due to climate change, floods and droughts have had devastating effects on human health by facilitating the spread of infectious illnesses including cholera, malaria, typhoid, dengue fever, and viral hepatitis A. Food instability, starvation, malnutrition, and a lack of potable water are only some of the indirect effects of flooding on health. Recently, one of the worst floods in history devastated Pakistan, affecting more than 333 million people along with a significant portion of the nation submerged. Malaria, dengue fever, and other ailments are on the rise in Pakistan, threatening to overwhelm the country’s healthcare infrastructure. There is an urgent need for preventative measures in Pakistan to cope with dreadful outbreaks.

Every year in Pakistan, heavy rainfall results in a surge in the outbreak of water-borne illnesses and viral epidemics. Due to Climatic variability floods, and droughts occur more often nowadays, which has severely impacted human health secondary to increased incidence of several infectious diseases such as Gastroenteritis, viral hepatitis A, yellow fever, cholera, malaria, typhoid fever, and dengue haemorrhagic fever. Similarly, a high incidence of acute conjunctivitis and transmittable eye infections has been reported during heavy rainfall and floods in Pakistan^1^.

The unavailability of a safe drinking water supply system, and poor sanitation, eventually led to water-borne disease outbreaks^2^. Such outbreaks result in a significant number of deaths^1^. Displacement of people from their homes inadvertently affects their mental health. Food shortage, starvation, malnutrition, and a lack of potable water are only some of the indirect effects of flooding on health. Children who lose their families become victims of illegal activities such as begging, child slavery, and adultery, and even get more exposure to sexual harassment^2^.

One of the most catastrophic floods in history hit Pakistan recently affecting 33 million people. It drowned one-third of the country underwater. More than 1300 people died since June 2022. The destructive flash floods have washed away millions of houses, crops, roads, and bridges along with hundreds of healthcare centres^3^.

Initially during flood mosquito breeding reduces, but post-flood stagnant water provides a perfect breeding place for malaria-causing mosquitos. It usually takes ~6–7 weeks for a malaria outbreak to occur^4^. The number of people infected with malaria globally reached ~241 million in 2020, which includes 5.7 million from Pakistan, with an estimated 627 000 fatalities^4^. Despite reporting a large number of malarial cases annually, Pakistan still lacks proper preventive approaches to deal with such outbreaks. This situation is further exasperated by the breakdown of healthcare services during the flooding situation, exposing affected people to the outbreak of various infectious diseases^5^.

Similarly, Post-flood stagnant water has resulted in the outbreak of another devastating disease; dengue. Dengue fever, along with its serious manifestations including dengue shock syndrome and dengue haemorrhagic fever has emerged as a significant public health problem after recent floods^6,7^. A tremendous increase in the incidence of dengue was experienced in the country, reporting a total of 1807 cases, 932 cases in July, and 875 cases in August alone^7,8^. These outbreaks of dengue and malaria along with other diseases had been distressing to the already exhausted healthcare system^1–3^. The destruction caused by the recent flood has exposed the inadequacies of our healthcare system. With merely 2% of the country’s GDP accounting for public health, it is evident that our current health system is unable to cope with any emergency^9^.

To face an adequate response to such humanitarian crises secondary to extreme climatic changes; there is a need for awareness in the general public to take preventive measures. This can be achieved via awareness programs, and campaigns through messaging and caller tune services. Government shall take mandatory prophylactic measures including chlorination of water, immunization, malaria prophylaxis, and handling of corpses. The use of mosquito sprays is not sufficient enough. In addition to larvicide, the bio-control by guppy fish which is a cheap and effective tool can be used to combat the dengue epidemic. It is imperative to upgrade surveillance to detect incursions of mosquitos and viruses in new areas and to apply viable approaches to mosquito control and disease prevention.

## Ethical approaval

Not applicable.

## Sources of funding

The author(s) received no financial support for the research, authorship, and/or publication of this article.

## Author contribution

Manuscript was written by O.A.S., M.R., M.E., and S.N. Review-editing was done by K.U., M.A.H., and S.O. Referencing and formatting was done by S.O.

## Conflicts of interest disclosure

The author(s) declared no potential conflicts of interest concerning the research, authorship, and/or publication of this article.

## Provenance and peer review

Not commissioned, externally peer-reviewed.
